# Supplementary material for: Magnetic Polyion Complex Micelles for Cell Toxicity Induced by Radiofrequency Magnetic Field Hyperthermia
Source: Nanomaterials (Basel). 2018 Dec 6;8(12):1014. doi: 10.3390/nano8121014 (PMC6316531; doi:10.3390/nano8121014)
Supplement: Supplementary file 1 [file nanomaterials-08-01014-s001.zip › SI Nanomaterials final.pdf]

nanomaterials

Supporting Information

Magnetic Polyion Complex Micelles for Cell Toxicity Induced by Radiofrequency Magnetic Field Hyperthermia

Vo Thu An Nguyen [1,2,3], Marie-Claire De Pauw-Gillet [4], Mario Gauthier [2,*] and Olivier Sandre [1,3*]

[1] Univ. Bordeaux, LCPO, UMR 5629, F-33600 Pessac, France; annguyenvothu@gmail.com
[2] Department of Chemistry, University of Waterloo, Waterloo, ON N2L 3G1, Canada; gauthier@uwaterloo.ca
[3] CNRS, Laboratoire de Chimie des Polymères Organiques, UMR 5629, F-33600 Pessac, France; olivier.sandre@enscbp.fr
[4] Mammalian Cell Culture Laboratory, GIGA-R, University of Liège, B-4000 Liège, Belgium; marie-claire.depauw@ulg.ac.be
* Correspondence: olivier.sandre@enscbp.fr; gauthier@uwaterloo.ca

1. Materials

Iron(III) chloride ($FeCl_3$, Sigma-Aldrich, anhydrous, powder, ≥ 99.99% trace metals basis), iron(II) sulfate heptahydrate ($FeSO_4{\cdot}7H_2O$, Sigma-Aldrich, ACS reagent, ≥ 99.0%), hydrochloric acid (HCl, Sigma-Aldrich, ACS reagent, 37%), ammonia (NH_4OH Sigma-Aldrich, 28-30% solution), and sodium hydroxide (NaOH, Sigma-Aldrich, pellets, semiconductor grade, 99.99% based on trace metals basis) were used as received. Milli-Q water (18.2 MΩ·cm) was obtained from a Milli-Q water purification system (Purelab Classic, Elga Lab Water). Linear poly(4-vinylpyridine) P4VP ($\overline{M}_n$ = 32,000 g·mol^{-1} and $\overline{M}_w$ = 65,000 g·mol^{-1}) was purchased from Aldrich. Ultrafiltration tubes (Amicon® Ultra-15 15 mL 100 kDa MWCO) were purchased from Dominique Dutscher SAS, France. Dulbecco modified Eagle medium (DMEM) with 4.5 g·L^{-1} glucose, sodium bicarbonate, without sodium pyruvate, sterile-filtered, suitable for cell culture, GlutaMAX, fetal bovine serum (FBS), and 0.5% trypsin solution 10× were purchased from Gibco, Grand Island, NY. Sodium pyruvate was obtained from Biowhittaker (Walkersville, MD). PBS 10× solution (with Ca^{2+} and Mg^{2+}), PBS 10× solution (Ca^{2+} and Mg^{2+} free), penicillin and streptomycin were purchased from Lonza. 3-(4,5-Dimethylthiazol-2-yl)-5-(3-carboxymethoxyphenyl)-2-(4-sulfophenyl)-2H-tetrazolium (MTS) was obtained from Promega (Madison, WI) for the cell viability tests. Paraformaldehyde (Sigma-Aldrich, powder 95%), Triton X-100 (Sigma-Aldrich, BioXtra, for molecular biology), bovine serum albumin (BSA), Alexa Fluor® 594 phalloidin (Life Technologies, 300 units), 4′,6′-diamidino-2-phenylindole (DAPI, Sigma-Aldrich 98.0%), and Vectashield HardSet Mounting Medium with DAPI (Vector laboratories) served for the cell internalization experiments

2. Synthesis of double hydrophilic block copolymer PAA-*b*-PHEA

Poly(tert-butyl acrylate) PtBA-Br macroinitiator. An atom-transfer radical polymerization (ATRP) procedure reported by Davis and Matyjaszewski[1] was adapted. Briefly, methyl 2-bromopropionate (0.368 mL, 3.3 mmol, 1 equiv), CuBr (0.252 g, 1.75 mmol, 0.53 equiv), PMDETA (0.363 mL, 1.74 mmol, 0.52 equiv), degassed acetone (5 mL), and *t*BA (20 mL, 140 mmol, 42.4 equiv) were added to an oven-dried round-bottomed flask and the reagent mixture was degased with 3 freeze-pump-thaw cycles. The green mixture was then placed in an oil bath at 60 °C for 2 h 40 min to reach $\bar{X}_n$ = 13, and 5 h 20 min to reach $\bar{X}_n$ = 27. The reactions were stopped by cooling the flask in liquid nitrogen and non-degased acetone (ca. 10 mL) was added. The solution was passed through a column (10 cm height × 1 cm diameter) of activated neutral alumina (Sigma-Aldrich, Brockmann I grade, 150-mesh size) using ca. 50 mL acetone as eluent. After removal of the organic solvent by

rotary evaporation, the polymer was dissolved in a minimum amount of methanol (ca. 10 mL) and further purified by dialysis in a 1,000 MWCO Spectra/Por® 7 regenerated cellulose bag against 1 L of methanol. The solvent was changed thrice within 24 h, and the polymer was collected by removal of the solvent under vacuum.

Silylation of 2-hydroxyethyl acrylate. A procedure reported by Mühlebach et al.[2] was adapted. The monomer HEA (75 mL, 0.652 mol, 1.0 equiv), dichloromethane (750 mL) and triethylamine (109.5 mL, 0.79 mol, 1.21 equiv) were added in a 2-L rbf and the solution was cooled to 0 °C in an ice bath. Trimethylsilyl chloride (91.5 mL, 0.72 mol, 1.1 equiv) was then added drop-wise, resulting in the formation of a white precipitate ($Et_3N{\cdot}HCl$). The solution was stirred in the ice bath overnight and allowed to reach room temperature gradually. The precipitate was removed by suction filtration (Whatman® filter paper, grade 4), the solvent was removed by rotary evaporation, and the liquid residue was filtered once more. The 2-trimethylsilyloxyethyl acrylate (HEATMS) was purified by dissolution in ethyl acetate (300 mL) and washing with water (300 mL) three times. Anhydrous $MgSO_4$ (ca. 5 g) was then added to the solution which was decanted after 15 min, and the solvent was removed under vacuum.

Synthesis of poly(*tert*-butyl acrylate)-*b*-poly(2-trimethylsilyloxyethyl acrylate) P*t*BA-*b*-P(HEATMS). P*t*BA-*b*-P(HEATMS) was synthesized by ATRP using either P(*t*BA)$_{13}$-Br ($\overline{M_n}$ = 1660) or P(*t*BA)$_{27}$-Br ($\overline{M_n}$ = 3460) as macroinitiator. The procedure reported here used P(*t*BA)$_{27}$-Br. In a 100-mL Schlenk flask were added CuBr (65 mg, 450 μmol, 1 equiv), anisole (10 mL), PMDETA (93 μL, 44.5 mmol, 0.99 equiv), and freshly distilled HEATMS (31.6 mL, 157 mmol, 350 equiv). The reaction mixture was stirred for 5 min before the addition of P*t*BA-Br (1.555 mg, 450 μmol, 1.0 equiv). The solution was then degased by three successive freeze-pump-thaw cycles, the flask was filled with N_2, and heated to 85 °C for 4 h 20 min to reach $\bar{X}_n$ = 60, and 19 h to reach $\bar{X}_n$ = 260. The reactions were stopped by submerging the flask in liquid nitrogen, before the addition of acetone (50 mL). The solution was passed through a column (15 cm height × 2 cm diameter) of activated neutral alumina (Aldrich-Sigma, Brockmann I grade, 150 mesh size) using acetone (100 mL) as eluent. After removal of the organic solvent by rotary evaporation, the polymer was dissolved in a minimum amount of methanol (ca. 30 mL) and further purified by dialysis in a 1,000 MWCO Spectra/Por® 7 regenerated cellulose bag against 1 L of methanol. The solvent was changed thrice within 24 h, and the polymer was collected after removal of the solvent under vacuum.

Hydrolysis of P*t*BA-*b*-PHEATMS. The procedure reported here was applied to P(*t*BA)$_{27}$-*b*-P(HEATMS)$_{260}$ to produce the corresponding PAA$_{27}$-*b*-PHEA$_{260}$. To a mixture of THF (83 mL) and CH_2Cl_2 (83 mL) was added P(*t*BA)$_{27}$-*b*-P(HEATMS)$_{260}$ (16.5 g, 81.9 mmol HEATMS units, 8.5 mmol *t*BA units, ca. 10% v/v). The flask was cooled in an ice-water bath with vigorous stirring and when the polymer was dissolved, H_2O (5 mL) was added to the solution followed by trifluoroacetic acid (TFA, 277 mL, 3.62 mol, 40 equiv) drop-wise. The solution was left to react for 24 h. The solvent was then removed under vacuum and the deprotected polymer was redissolved in ethanol (EtOH, 30 mL). Solvent exchange was performed by dialysis in a 1,000 MWCO Spectra/Por® 7 regenerated cellulose bag against EtOH (2 L) for 24 h, followed by EtOH/H_2O (50/50 v/v, 2 L) for 24 h, and finally against 6 changes of H_2O (5 L) for 72 h. To accelerate the dialysis, the pH of the H_2O used in the last step was maintained at pH 7 by the addition of NaOH solution. The clear solution obtained was collected and stored at 4°C.

Fluorescently labeled block copolymer. The labeled PAA$_{27}$-*b*-PHEA$_{260}$ copolymer (hereinafter referred to as PAA$_{27^*}$-*b*-PHEA$_{260}$) was prepared via amidation of a small fraction of the carboxylic groups in the PAA block with fluoresceinamine, isomer I (FA, Sigma-Aldrich, 75%), using 1-ethyl-3-(3-dimethylaminopropyl) carbodiimide hydrochloride (EDC, Sigma-Aldrich, ≥ 99.0%). A water dispersion of PAA$_{27}$-*b*-PHEA$_{260}$ (50 mg, 0.162 μmol BC, 43.6 μmol of COOH groups) was prepared while fluoresceinamine (FA, Sigma-Aldrich, 75%, 3.35 mg, 9.65 μmol, FA/COOH = 6/27 mol/mol) was dissolved in DMF (2 mL). The two solutions were combined and vigorously stirred for 10 minutes before 1-ethyl-3-(3-dimethylaminopropyl) carbodiimide hydrochloride (EDC, Sigma-Aldrich, ≥ 99.0%, 67.73 mg, 436 μmol, EDC/COOH = 270/27) was added to create the highly reactive O-acylisourea intermediate. The mixture was allowed to react for 24 h before the product

was dialyzed against deionized water for 5 d with 15 changes of water. The PAA_{27}*-*b*-$PHEA_{260}$ copolymer was collected and stored at 10 °C. The fluorescence emission of the block copolymer was monitored on a UV/Vis/Fluorescence SpectraMax® M2e Multimode Microplate Reader.

3. Characterization of MPIC micelles

Dynamic light scattering (DLS) was performed in triplicate using disposable PS cuvettes or quartz cuvettes on a Zetasizer Nano ZS90 (Malvern Instruments) equipped with a 4 mW He-Ne laser operating at 633 nm and 25.0 °C, at a scattering angle of 90°. The Z-average hydrodynamic diameter (Dh) and polydispersity index (PDI) were calculated using the standard 2nd order cumulant fitting procedure of the correlograms of the scattered light intensity.

Atomic force microscopy (AFM) images were recorded in the tapping mode in air on a Veeco Dimension Icon System equipped with a Nanoscope V controller. The probes used were Olympus® AC160TS-R3 micro cantilevers (spring constant 26–56 N/m, resonance frequency 300–399 kHz, curvature radius 8–10 nm). The scan rate was typically between 0.7 and 1.2 Hz, at a scan angle of 0°, acquiring 512 samples/line. For sample preparation, a 20 µL aliquot of MPIC micelle dispersion (0.05–0.1 mg/mL in water) was deposited on the freshly cleaved mica substrate and spin-coated at 3000 rpm for 180 s under ambient conditions.

Transmission electron microscopy (TEM) images were obtained on a Hitachi H7650 electron microscope operating at 80 kV. Samples were prepared by depositing 0.7 µL of the MNP aqueous solution onto a copper grid (200-mesh coated with holey carbon film) and blotting the excess after 5 min. The grids were let to dry overnight under a fume hood. The metal-free samples were stained by exposing the grids to iodine vapors for 60 min.

Thermogravimetric analysis (TGA) was conducted on a Q500 (Q Series™ TA Instrument) following the following program: 1) Select N2 gas; 2) Equilibrate at 30 °C; 3) Ramp at 10 °C/min to 140 °C; 4) Isothermal for 25 min; 5) Ramp at 10 °C/min to 470 °C; 6) Isothermal for 20 min; 7) Select air gas; 8) Ramp at 10 °C/min to 800 °C; 9) Equilibrate at 200 °C.

4. Biocompatibility assessment

Cell cultures. The fibroblast-like L929 cells derived from normal subcutaneous areolar and adipose tissue of a 100-day old male C3H/An mouse[3] was purchased from European Collection of Cell Cultures (85011425, ECACC, UK). L929 cells were cultured in DMEM supplemented with 10 vol% of heat-inactivated fetal bovine serum (FBS), 1 vol% of antibiotics (10,000 units of penicillin and 10,000 units of streptomycin/mL), 1 vol% sodium pyruvate and 1 vol% GlutaMAX. The cells were maintained at 37 °C in a 5% CO_2 humidified incubator. After rinsing with PBS (Ca^{2+}/Mg^{2+} free) solution to remove the serum, the cells were detached with trypsin solution 10× diluted 10-fold with PBS (Ca^{2+}/Mg^{2+} free) solution. The PBS solution used contained Ca^{2+}/Mg^{2+} unless otherwise noted (e.g., Ca^{2+}/Mg^{2+} free).

Cytotoxicity assessment. After detachment, the L929 cells were seeded in a 96-well plate at a density of $2×10^3$ cells/well, and grown in DMEM complete medium in a humidified incubator (5 vol% CO_2) at 37 °C for 24 h prior to treatment. The MPIC micelle solutions with different final concentrations in the culture medium (1250, 700, 140, 70, 28, and 14 µg of Fe_3O_4/mL) were prepared in a mixture of 10% PBS solution and 90% DMEM complete medium before cell treatment. The cells were exposed to MPIC micelle solutions and incubated for 48 h. The medium was removed and the cells were rinsed with PBS solution (100 µL/well). A 120 µL aliquot of MTS solution (containing 20 µL of MTS solution and 100 µL of PBS solution) was then added into each well. The plates were incubated at 37 °C for 120 min. The absorbance at 490 nm was measured on a PowerWave X (BioTek Instrument Inc.) micro-plate UV-VIS spectrometer. The positive controls were cells incubated with the mixture of 10% PBS solution and 90% DMEM complete medium alone. The results were expressed as the percentage of metabolic activity of treated cells relatively to untreated cells (control, 100% viability). Independent experiments were performed 3 times with 4 replicates per condition.

Cell internalization study by confocal laser scanning microscopy. The L929 cells were seeded on the surface of a sterile glass coverslip placed in the well of a 12-well plate at a density of $5×10^4$

cells/2 mL/well and allowed to attach and to grow at 37 °C in a 5% CO_2 humidified incubator. The fluorescently labeled MPIC* micelle solution (G1@Fe_3O_4@PAA*$_{27}$-*b*-PHEA$_{260}$ f = 0.5; 140 µg Fe_3O_4/mL) was prepared in a mixture of 10% PBS solution and 90% DMEM complete medium before cell treatment. After 24 h of cell growth the DMEM complete medium was replaced with 2 mL of MPIC* micelle suspensions. From this point the plate was protected from light exposure by wrapping in aluminum foil when it was not in use. After 24 h of exposure to the nanoparticles, the suspension was removed and the cells were rinsed twice with PBS solution to completely remove not untaken nanoparticles. The cells were then fixed with 2 mL of paraformaldehyde cross-linking agent (4 vol%) diluted in PBS solution (4 °C, 20 min). The fixative solution was removed and the cells were washed twice with PBS solution. Permeabilization of cell membranes was performed by treating the cells with 2 mL of 0.5 vol% Triton X-100 in PBS solution (4 °C, 20 min). The Triton X-100 solution was removed and the cells were rinsed twice with PBS solution. The protein-binding sites on the membranes were blocked by incubating with 1 vol% BSA in PBS solution (2 mL, 37 °C, 30 min). The blocking agents were removed and the cells were washed twice with PBS solution before 2 mL of 1.67 vol% of Alexa Fluor® 594 phalloidin in PBS solution was added to label the *F*-actin cytoskeleton of the fixated cells. The actin staining was allowed to occur for 1 h at 37 °C in the humidified incubator before the cells were washed twice with PBS solution. The cell nuclei were then stained with 2 mL of 0.025 vol% of DAPI in PBS solution (10 min, 4 °C). The staining solution was removed and the cells were rinsed twice and stored in 0.05 vol% of Tween-20 in PBS solution, to improve the assay sensitivity by reducing background interference. The coverslips were picked up, allowed to dry for 2 h, turned over and permanently mounted to the glass slide using one drop of Vectashield HardSet Mounting Medium with DAPI (refractive index 1.452).

Analysis of the control and treated L929 cells was performed using a Leica TCS SP5 confocal laser scanning microscope equipped with a 405 nm diode UV laser for blue fluorescent labeling (DAPI filter set, *Ex*: 405 nm, *Em*: 461 nm), an argon laser for green labeling (Green Fluorescent Protein filter set, *Ex*: 488 nm, *Em*: 525 nm) and DPSS 561 laser for red fluorescent labeling (Alexa filter set, *Ex*: 581 nm, *Em*: 609 nm). The scan speed was 400 Hz. The used objective was a Leica HCX PL APO 63x magnification /1.3 numerical aperture objective lens embedded in 100% glycerol (refractive index 1.46). Images were taken at 1024 pixel × 1024 pixel resolution. The photomultiplier gain and offset configurations were set up on control cells so as to correct the images for green auto-fluorescence. A *Z*-stack of 50 frames covering a depth of 10 µm was recorded for assessment of the NP distribution across the section. The images were processed using the ImageJ (Image Processing and Analysis in Java) and ICY (an open community platform for bioimage informatics) software.

Cell internalization studies using TEM. The L929 cells were seeded in a 6-well plate at a density of 2.5×10^5 cells/2 mL/well and allowed to grow at 37 °C in a 5% CO2 humidified incubator. The MPIC micelle suspension (140 µg of Fe_3O_4/mL) was prepared in a mixture of 10% phosphate buffered saline (PBS) 1× 150 mM pH7.4 solution and 90% DMEM complete medium before cell treatment. After 24 h the DMEM complete medium was replaced with 2 mL of MPIC micelle suspension. After 24 h of exposure to the NPs, the suspension was removed and the cells were rinsed twice with PBS (Ca^{2+}/Mg^{2+} free) 1× solution to completely remove non-internalized NPs. After being detached with trypsin solution 10× diluted 10-fold in PBS (Ca^{2+}/Mg^{2+} free) solution, the cells were centrifuged at 200 rcf for 5 min. The trypsin solution was carefully removed and the cells were fixed with a 2.5% glutaraldehyde solution diluted in PBS 1× solution at 20 °C for 30 min. The cells were then centrifuged at 200 rcf for 5 min, the fixative was removed, and the cells were washed and redispersed in H2O.

For observation by TEM, the cells were first placed in vials where they were post-fixed for 30 min in 1% OsO_4, washed with water, and air-dried. Epoxy resin (Epon 812, AGAR 100) was directly cast and polymerized in the vial. To facilitate sectioning, the cell resin was isolated and cut in two halves before re-embedding in resin prepared in flat silicone rubber molds. Sections with a thickness estimated at approximately 200 nm were cut with a glass knife. Observation was made without further staining on a Philips 301 electron microscope at an accelerating voltage of 80 kV.

Cell internalization studies using Fluorescence-Activated Cell Sorting (FACS). The L929 cells were seeded in a 24-well plate at a density of 4×10^5 cells/well and allowed to grow at 37 °C in a 5% CO_2 humidified incubator. For the kinetics experiments, the fluorescently labeled MPIC* micelle solution (140 µg of Fe_3O_4 content/mL) was prepared in a mixture of 10% PBS solution and 90% DMEM complete medium before cell treatment. For concentration effect experiments, the MPIC* micelle solutions were also prepared in a mixture of 10% PBS solution and 90% DMEM complete medium at various concentrations (1.4, 14, 28, 70 and 140 µg of Fe_3O_4/mL). After 24 h incubation, the DMEM complete medium was replaced with 1 mL of MPIC* micelle suspension. The magnetic nanoparticle exposure time periods for cells in the kinetics experiments were 1, 4, 8, 12 and 24 h, while all the cells used in the concentration tests were exposed to the MPIC* micelles for 24 h. After desired exposure to the NPs, the suspension was removed and the cells were rinsed twice with PBS 1× (Ca^{2+}/Mg^{2+} free) solution to remove non-internalized NPs. After being detached with trypsin 10× solution 10-fold diluted in PBS 1× (Ca^{2+}/Mg^{2+} free) solution, the cells were centrifuged at 200 rcf for 5 min. The trypsin solution was removed before the cells were fixed with a 4% solution of paraformaldehyde diluted in PBS 1× solution at 20 °C for 30 min. The cells were then centrifuged at 200 rcf for 5 min, the fixative was removed, and the cells were washed and redispersed in PBS 1× solution. The control cells were prepared using the same protocol but without being exposed to the fluorescently labeled magnetic NPs. Quantitative studies were performed using the FACS technique on a FACScan system (FACS, Becton-Dickinson). The mean fluorescence intensity (MFI) of 10,000 treated L929 cells was compared with that of 10,000 untreated cells. The percentage of fluorescence-positive cells per 10,000 cells was also reported. The mean values are reported with the standard deviations from three independent experiments.

5. Supporting figures

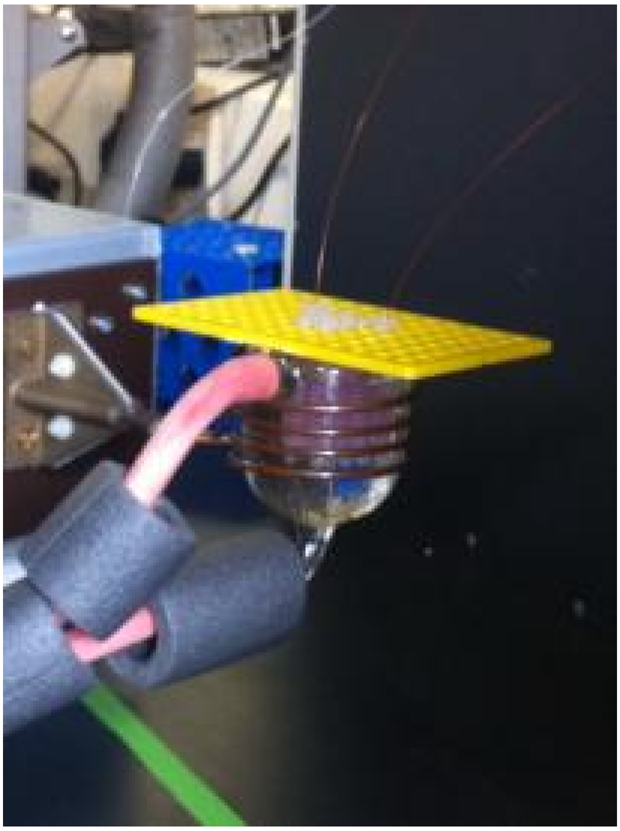

Figure S1. Photography of MFH setup: 6 plastic 0.4 mL NMR tubes containing the cells are placed in a 37°C water bath along the axis of a 4-turn copper coil of 10 mm inter-loop spacing and 55 mm

diameter. The double-wall glass jacket insured thermal insulation from the inducting coil and enable thermo-regulation through water circulation at 37°C (Huber Polystat™ CC, Offenburg, Germany)

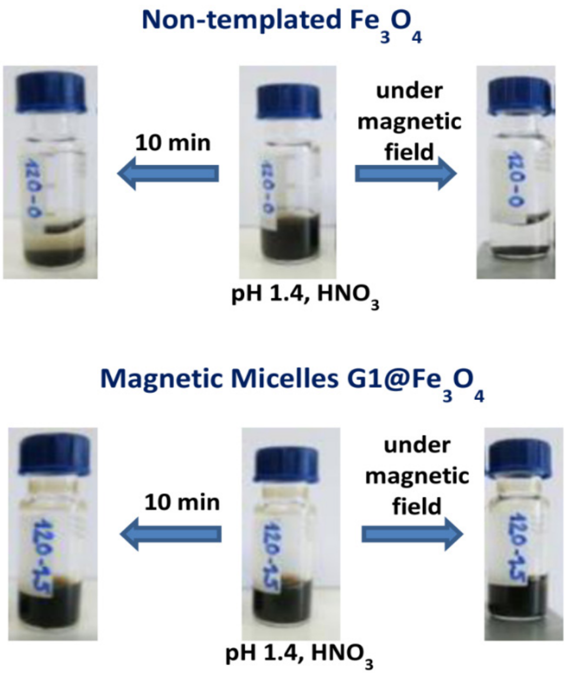

Figure S2. Photography of sample vials: Improved colloidal stability of templated G1@Fe_3O_4 ferrofluid as compared with non-templated Fe_3O_4 ferrofluid.

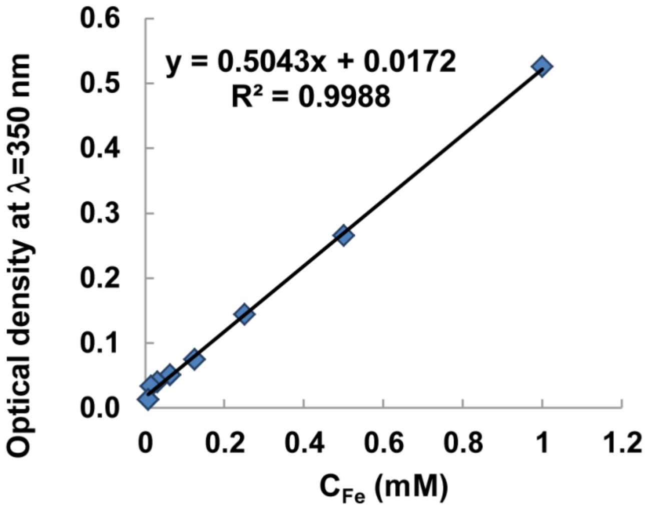

Figure S3. Calibration curve for iron concentrations in 5 M HCl generated using the absorbance at λ = 350 nm (path length l = 2 mm). The titrating solutions used to build this curve were diluted from a $FeCl_3$ solution certified at 44.7 wt% ($[Fe^{3+}]$=4.09 mol·L^{-1}).

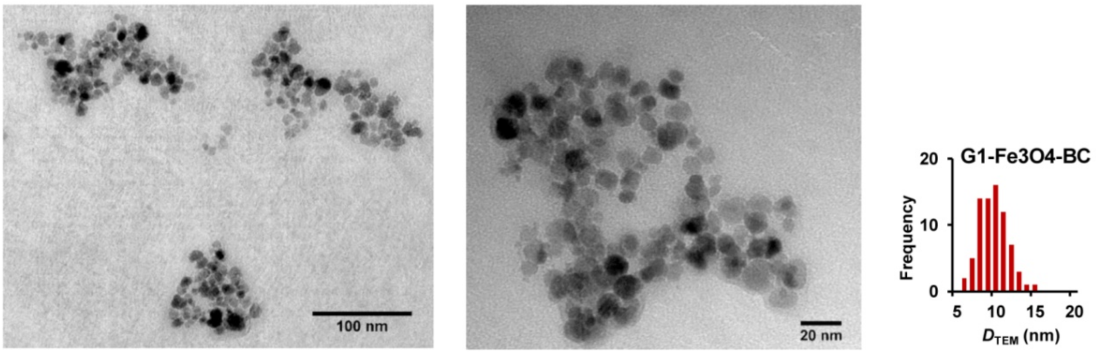

Figure SI-4. TEM images of MPIC micelles showing Fe_3O_4 crystallites in $G1@Fe_3O_4@PAA_{27}$-*b*-$PHEA_{260}$ $f = 0.5$.

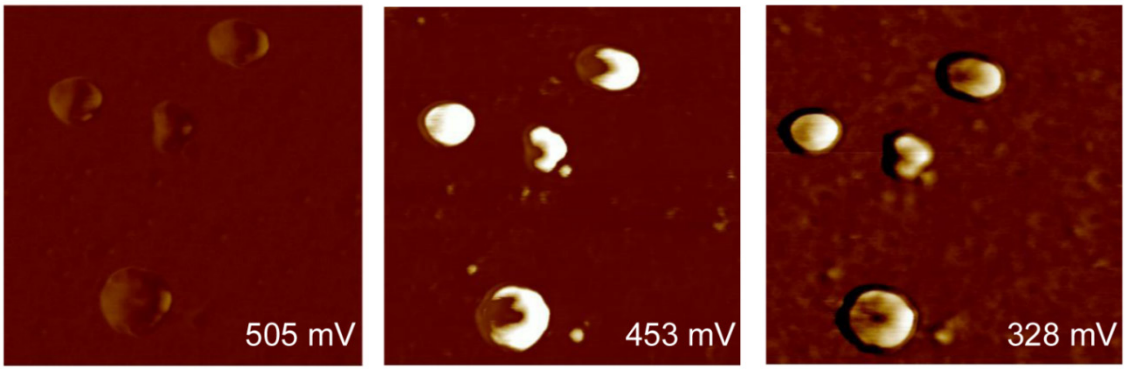

Figure S5. AFM images of MPIC micelles at various amplitude set points (the lower the value in mV, the higher the average deflection of the cantilever and thus the exerted force). The scale of all images is 500 × 500 nm^2.

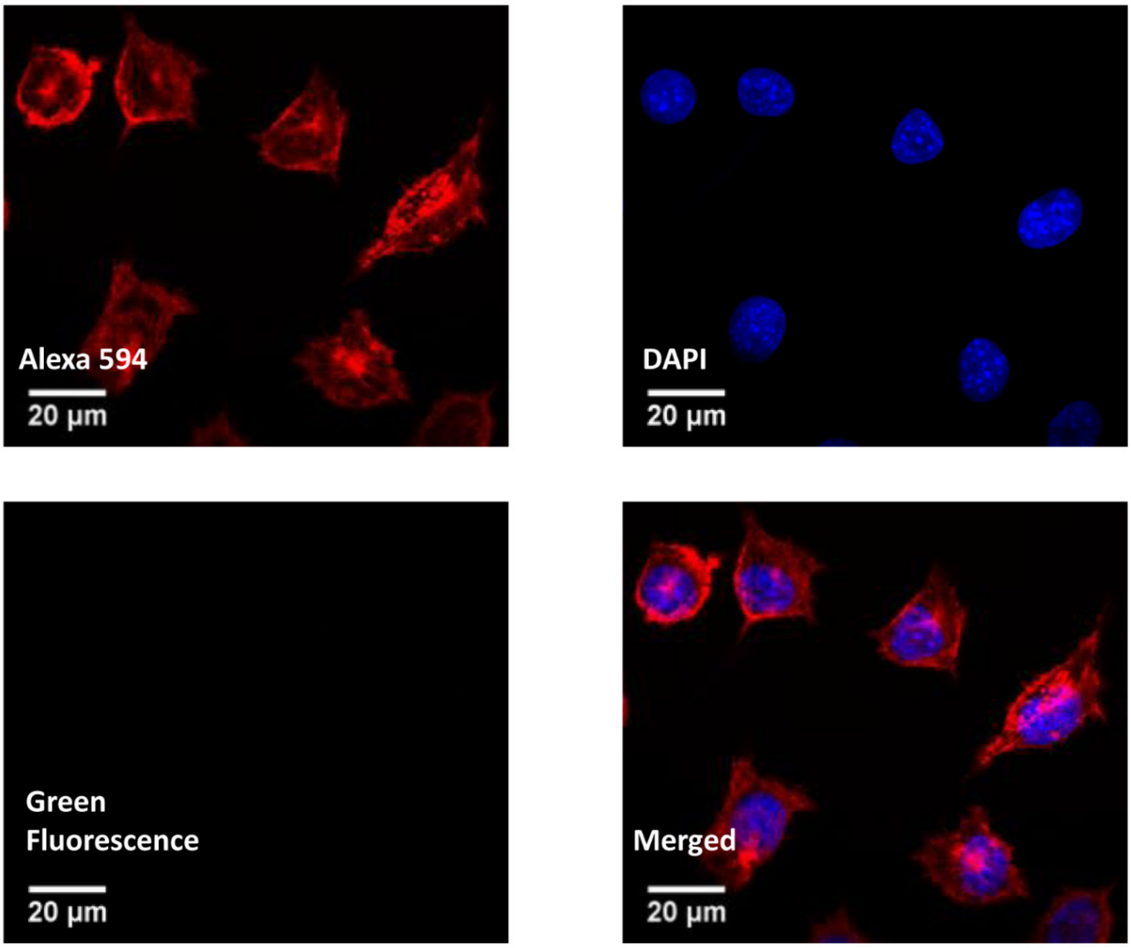

Figure S6. CLSCM images of L929 control cells untreated with MPIC micelles, after 24 h of incubation with pure medium.

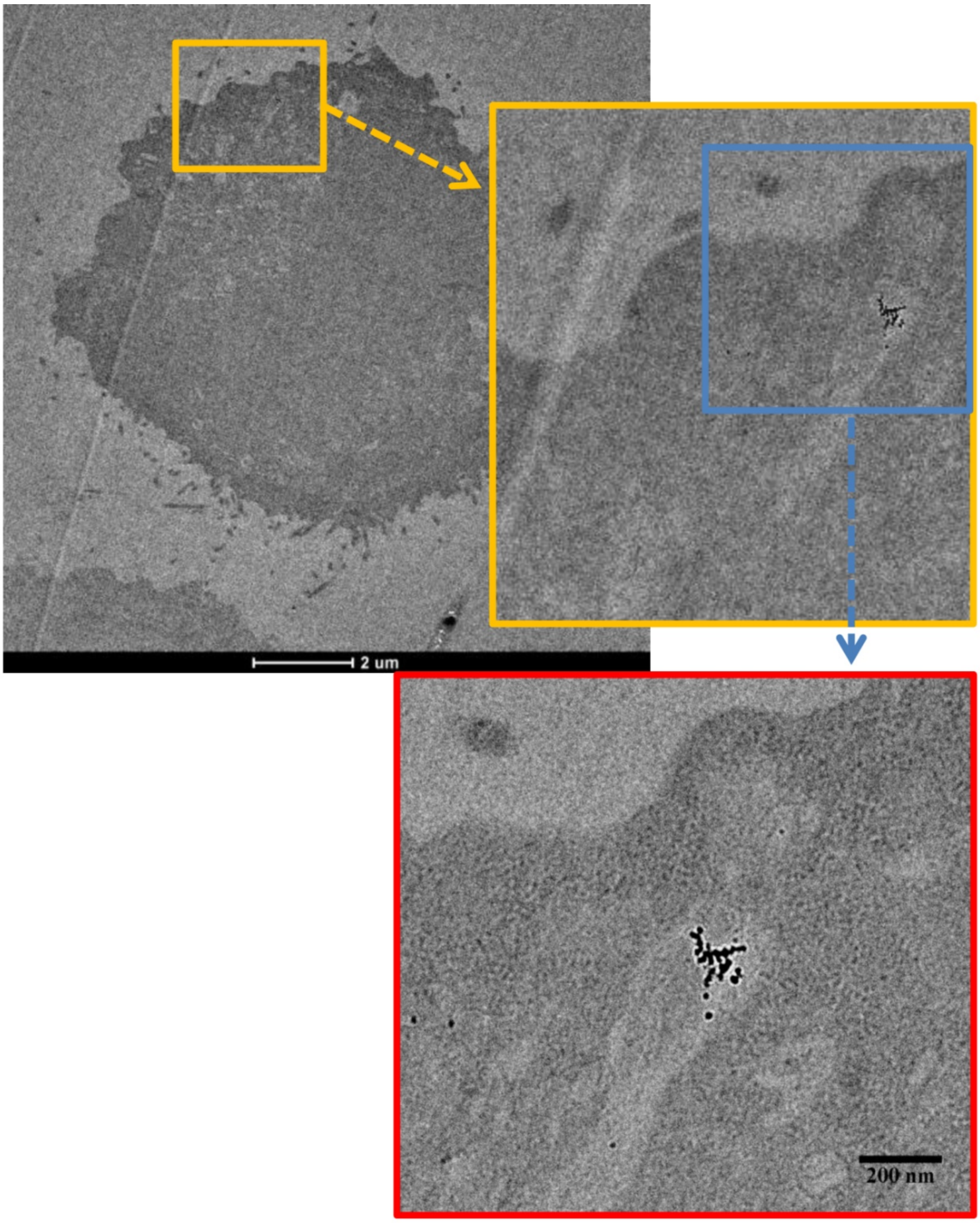

Figure S7. **TEM images of L929 cells treated with MPIC micelles** (140 μg Fe_3O_4/mL, 24 h incubation), inset showing iron oxide MNPs internalized as endosome inside the L929 cell.

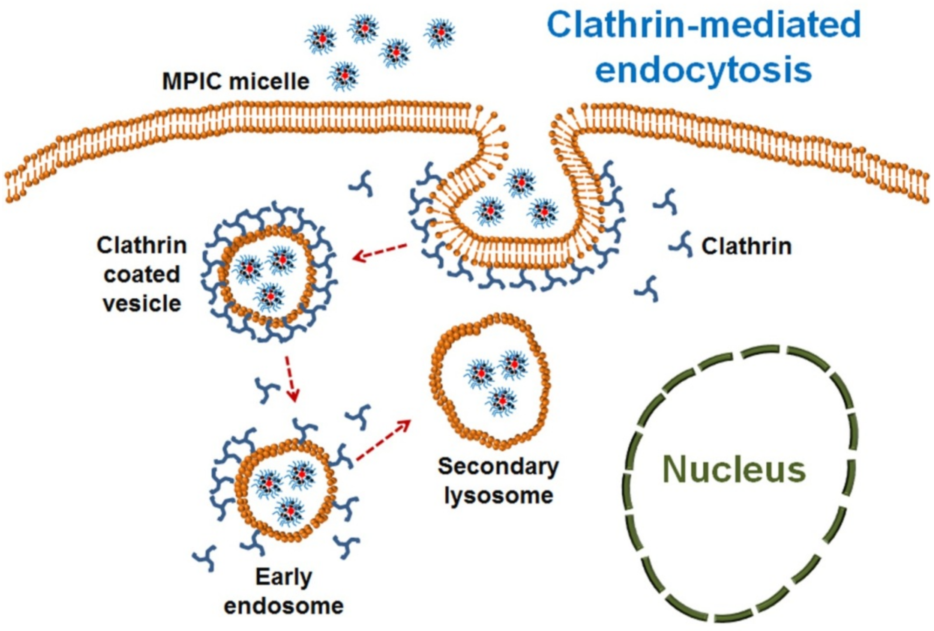

Figure S8. Proposed mechanism of clathrin-mediated endocytosis of MPIC micelles into the L929 cells.

1. Davis, K.A.; Matyjaszewski, K. Atom Transfer Radical Polymerization of tert-Butyl Acrylate and Preparation of Block Copolymers. *Macromolecules* **2000**, *33*, 4039-4047, doi:10.1021/ma991826s.
2. Mühlebach, A.; Gaynor, S.G.; Matyjaszewski, K. Synthesis of Amphiphilic Block Copolymers by Atom Transfer Radical Polymerization (ATRP). *Macromolecules* **1998**, *31*, 6046-6052, doi:10.1021/ma9804747.
3. LifeTechnologies. L929 Cells. Cell Line database: LifeTechnologies: 2014; Vol. Technical resources/Cell Lines/L/L929 Cells.

© 2018 by the authors. Submitted for possible open access publication under the terms and conditions of the Creative Commons Attribution (CC BY) license (http://creativecommons.org/licenses/by/4.0/).
