# Supplementary figures and images for: Magnetic Polyion Complex Micelles for Cell Toxicity Induced by Radiofrequency Magnetic Field Hyperthermia
Source: Nanomaterials (Basel). 2018 Dec 6;8(12):1014. doi: 10.3390/nano8121014 (PMC6316531; doi:10.3390/nano8121014)

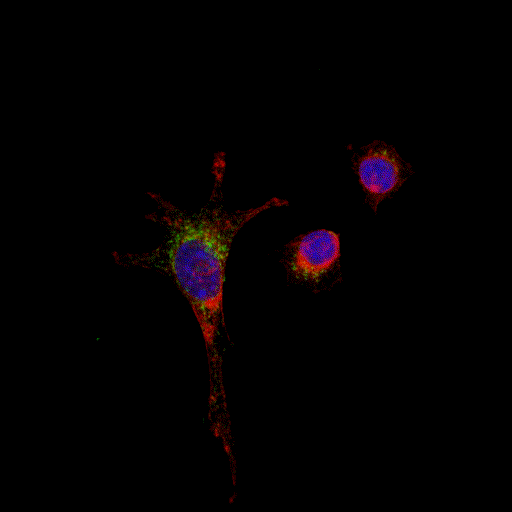

Supplement: Supplementary file 1 [file nanomaterials-08-01014-s001.zip › 3 channel CLSM video.gif]
